# Synthesis and Characterization of Two Sulfonated Resorcinarenes: A New Example of a Linear Array of Sodium Centers and Macrocycles

**DOI:** 10.3390/molecules20069915

**Published:** 2015-05-28

**Authors:** Edilma Sanabria, Miguel Ángel Esteso, Adrián Pérez-Redondo, Edgar Vargas, Mauricio Maldonado

**Affiliations:** 1 Departamento de Química, Universidad de los Andes, Cr. 1 No. 18A 10, Bogotá 111711, Colombia; E-Mails: e.sanabria84@uniandes.edu.co (E.S.); edvargas@uniandes.edu.co(E.V.); 2 U.D. Química Física, Universidad de Alcalá, Alcalá de Henares 28871, Spain; E-Mail: miguel.esteso@uah.es; 3 Departamento de Química Orgánica y Química Inorgánica, Universidad de Alcalá, Alcalá de Henares 28871, Spain; E-Mail: adrian.perez@uah.es; 4 Departamento de Química, Facultad de Ciencias, Universidad Nacional de Colombia, Sede Bogotá, Cr. 30 No. 45-03, Bogotá 111321, Colombia

**Keywords:** sulfonation, water soluble resorcinarene, cone conformation

## Abstract

Two sulfonated resorcinarenes were synthesized by reacting *C*-tetra(butyl)resorcinarene or *C*-tetra(2-(methylthio)ethyl)resorcinarene with formaldehyde in the presence of sodium sulfite. Their structures were determined via FT-IR, ^1^H-NMR, ^13^C-NMR and mass spectrometry. Thermal gravimetric analyses of the derivatives were also carried out and revealed the presence of water molecules in the solid state. The sulfonated product of *C*-tetra(butyl)resorcinarene was characterized by an X-ray crystal structure determination. The asymmetric unit contains eight molecules of water and two of acetone, and analysis indicated that sulfonated resorcinarene prefers a cone configuration (*rccc* conformation) in the solid state. In the crystal array, classical hydrogen bond interactions O-H···O and intermolecular contacts were observed. In the crystal packing, a linear array of capsules of sulfonated resorcinarenes was generated for a chain of sodium atoms and sulfonate groups.

## 1. Introduction

Resorcinarenes are macromolecules with four resorcinol rings linked by methylene bridges. They have an important place in supramolecular chemistry and are of interest in different fields of study, with applications in chemical separations [1,2,3], voltammetric sensors [4], dendrimer synthesis [5,6], dyeing of fibers [7,8], as NMR solvating agents [9], chemical receptors for molecules and ions [10,11,12], absorption of heavy metal ions [13] and the provision of easy access to cavitands and carcerands in synthesis [14]. Resorcinarenes are synthesized by the acid-catalyzed cyclocondensation of resorcinol with aldehydes [15,16] and can be functionalized in various ways: at the methylene bridge of the macrocyclic system during the acid-catalyzed condensation reaction by choosing the aldehyde as a starting material (modification in the lower rim) or via post-synthetic modification of the phenolic hydroxyl groups and/or the free position between hydroxyl groups of resorcinol rings (modification in the upper rim). The synthesis of these derivatives is essential for modulating their conformation via electronic and steric effects, including weak interactions. In the present paper, we describe the synthesis of two novel sulfonated resorcinarenes, which were characterized via FT-IR, ^1^H-NMR, ^13^C-NMR, mass spectrometry and TG analysis, as well as via an X-ray crystallographic determination of one of them.

## 2. Results and Discussion

**Scheme 1:**
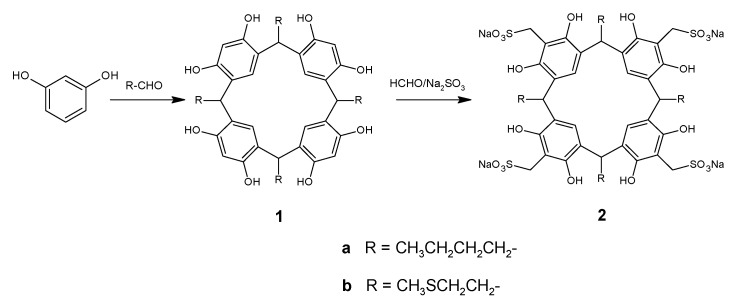
Synthetic route to the sulfonated resorcinarenes.

The first step involved the preparation of *C*-tetra(butyl)resorcinarene (**1a**) and *C*-tetra (2-(methylthio)ethyl)resorcinarene (**1b**) through the acid-catalyzed cyclocondensation of resorcinol with an aldehyde (valeraldehyde and 3-methylthiopropanaldehyde, respectively) in a 50:50 mixture of ethyl alcohol and water at 75 °C in a manner similar to that described in the literature [17] (Scheme 1). The products were purified by means of recrystallization. These derivatives were characterized using spectral techniques, including FT-IR, ^1^H-NMR, ^13^C-NMR and mass spectrometry (see the Experimental Section). Compound **1a** had been previously synthesized by other authors [18], and the spectroscopic data agreed with those reported by us. The FT-IR spectrum for **1b** is in agreement with the organic functionalities present in the structure of the compound, as it reveals hydroxyl group stretches at 3330 cm^−1^ (O-H) and 1160 cm^−1^ (C-O), whereas the C-S of the thioether group is observed at 605 cm^−1^; bands of the alkyl substituent and the aromatic ring are also observed. The ^1^H-NMR spectrum showed resonance signals for the aromatic hydrogen atoms (δ = 7.24 and 6.39), the methylene bridge fragments (δ = 4.50) and the thioether moieties (δ = 2.47 and 2.13). The attributions of the signals in the ^13^C-NMR spectra were based on the analysis of bidimensional techniques and are shown in the Experimental Section. The molecular ion in the mass spectrum was also consistent with the assigned structure for **1b**.

Sulfonation of *C*-tetra(butyl)resorcinarene (**1a**) and *C*-tetra(2-(methylthio)ethyl)resorcinarene (**1b**) was done by direct reaction with formaldehyde solution and sodium sulfite in water at 90–95 °C [19] (Scheme 1). Compound **2a** was obtained as a pale yellow solid, UV_λmax_ (H_2_O) 295 nm, and the molecular weight was determined via ESI-MS (1154.20 g/mol). The FT-IR spectrum of **2a** showed sulfonate group (1044 cm^−1^ and 773 cm^−1^), aromatic ring (1609 cm^−1^), alkyl chain (2929 cm^−1^) and hydroxyl group (3421 cm^−1^) absorptions. The ^1^H-NMR spectrum displayed characteristic signals of butyl chains (1.09, 1.45, 1.60 and 2.35 ppm), a methylene bridge fragment between the aromatic rings (4.60 ppm), a methylene bridge fragment between the aromatic rings and the sulfonate group (4.41 ppm) and the aromatic hydrogen of a pentasubstituted resorcinol unit (7.39 ppm). The carbon signals in ^13^C-NMR were unambiguously assigned through 1D- and 2D-NMR experiments, including HMQC and HMBC.

Compound **2b** was obtained as a pale yellow solid, UV_λmax_ (H_2_O) 288 nm, and the molecular weight was determined via ESI-MS (1193.24 g/mol). The ^1^H- and ^13^C-NMR data of **2b** were similar to those of **2a**, except that **2b** showed signals for the thioether chain at 2.09 ppm (-S-CH_3_), 2.43 ppm, 2.49 ppm and 4.39 ppm for the methylene bridge. The NMR spectroscopic analysis showed the highly symmetrical structure of sulfonated resorcinarenes in solution. Thus, it can be presumed that the preferred conformations in solution of **2a** and **2b** are more likely to be the cone ones (*rccc* conformation).

Thermal analysis was performed in order to establish the number of water molecules and in order to elucidate the stoichiometry in sulfonated resorcinarenes. The results are shown in Table 1.

**Table 1:** Thermal behavior data for sulfonated resorcinarenes.

Resorcinarene	Step	Temperature Range (°C)	Δm_exp_ (%)	Δm_calc_ (%)
**2a**	1	85–140	4.40	4.38
	2	270–350		
**2b**	1	97–147	4.39	4.33
	2	267–347		

**Figure 1:**
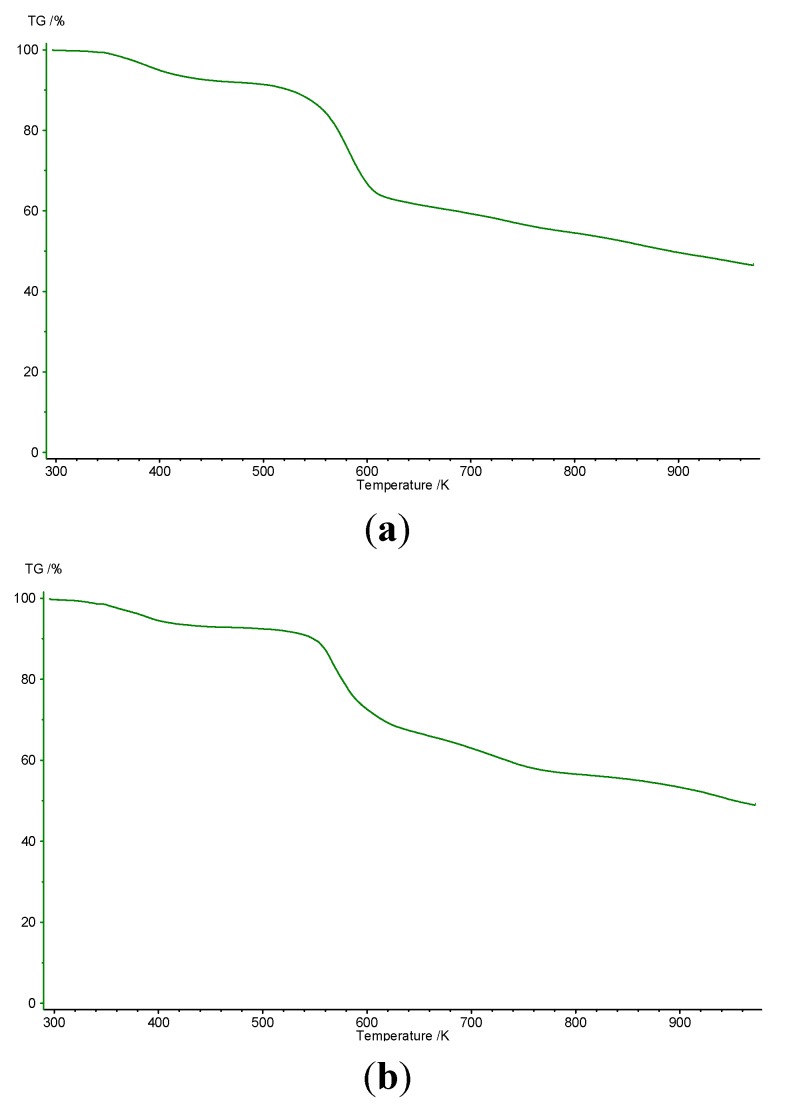
Thermograms of sulfonated resorcinarenes: (**a**) Compound **2a**; (**b**) Compound **2b**.

Sulfonated resorcinarene **2a** decomposes in two steps up to 700 °C. The elimination of three water molecules occurs between 85 and 140 °C in the first step. The next step corresponds to the oxidative degradation of resorcinarene, and it occurs between 270 and 350 °C (Figure 1a). For **2b**, the water molecules are released at 97 °C. This step is also followed by oxidative degradation of the resorcinarene at 267 °C. On the basis of the above data, the proposed number of water molecules in the two sulfonated resorcinarenes is three (Figure 1b).

These results are in agreement with previous studies for other resorcinarene systems, which show that molecules, such as water, alcohol, pyridine and dimethylformamide can generate solvates [20]. Therefore, most of the solid structures of resorcinarenes reported so far have one to three solvent molecules.

**Figure 2:**
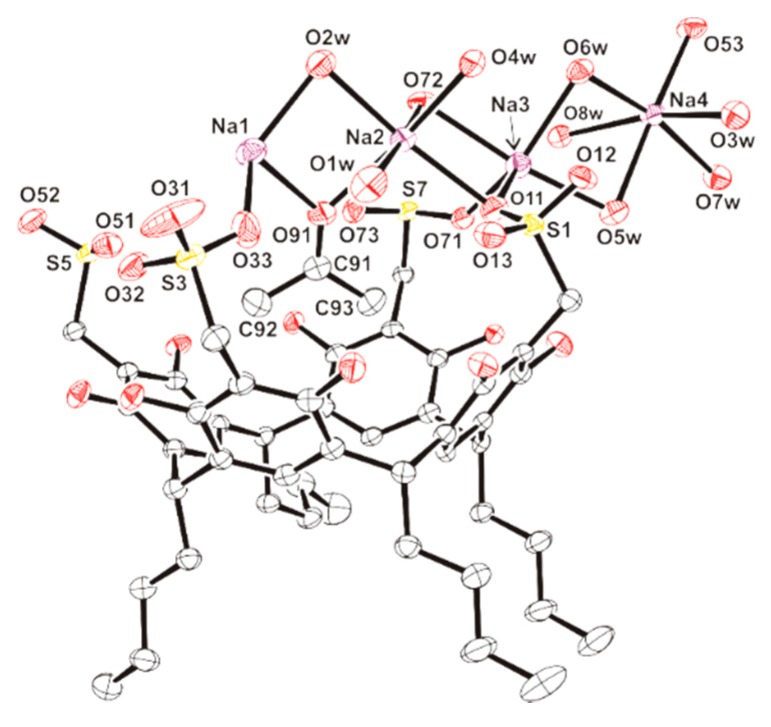
Asymmetric unit of the crystal structure for **2a** (thermal ellipsoids at the 50% probability level). Hydrogen atoms and molecules of acetone are omitted for clarity. Symmetry transformations: (a) *x*, *y* + 1, *z*.

Our attempts to grow crystals for Compound **2b** were unsuccessful, whereas crystals suitable for a single-crystal X-ray diffraction analysis for Compound **2a** were obtained from a water-acetone (1:1) solution. Compound **2a** crystallizes with eight molecules of water and two molecules of acetone as a one-dimensional coordination polymer. These crystals decompose when they are exposed to air, very likely due to the loss of solvent, but they are stable in solution. The asymmetric unit of **2a** is shown in Figure 2, while the values of both the selected lengths and the angles are given in Table 2.

**Table 2:** Selected lengths and angles for **2a**.

**Atoms**	**Length (Å)**	**Atoms**	**Length (Å)**
C(14)···C(54)	5.198(6)	C(20)···C(60)	7.152(5)
C(34)···C(74)	5.162(4)	C(40)···C(80)	7.249(5)
Na(1)-O(33)	2.281(3)	Na(1)-O(91)	2.375(3)
Na(1)-O(1w)	2.863(4)	Na(1)-O(2w)	2.359(3)
Na(1)-O(3w)a	2.313(3)	Na(1)-O(7w)a	2.342(3)
Na(2)-O(11)	2.414(3)	Na(2)-O(91)	2.337(3)
Na(2)-O(1w)	2.729(4)	Na(2)-O(2w)	2.401(3)
Na(2)-O(4w)	2.350(3)	Na(2)-O(72)	2.327(3)
Na(3)-O(11)	2.386(3)	Na(3)-O(71)	2.349(3)
Na(3)-O(5w)	2.370(3)	Na(3)-O(6w)	2.434(3)
Na(3)-O(8w)	2.887(3)	Na(3)-O(72)	2.514(3)
Na(4)-O(3w)	2.355(3)	Na(4)-O(5w)	2.472(3)
Na(4)-O(6w)	2.363(3)	Na(4)-O(7w)	2.368(3)
Na(4)-O(8w)	2.495(3)	Na(4)-O(53)	2.369(3)
**Atoms**	**Angles (deg)**	**Atoms**	**Angles (deg)**
O(33)-Na(1)-O(2w)	132.1(1)	O(33)-Na(1)-O(91)	94.5(1)
O(33)-Na(1)-O(1w)	60.6(1)	O(33)-Na(1)-O(3w)a	95.7(1)
O(33)-Na(1)-O(7w)a	126.0(1)	O(91)-Na(1)-O(1w)	69.6(1)
O(91)-Na(1)-O(2w)	86.9(1)	O(91)-Na(1)-O(3w)a	169.4(1)
O(91)-Na(1)-O(7w)a	87.6(1)	O(1w)-Na(1)-O(2w)	75.5(1)
O(1w)-Na(1)-O(3w)a	118.2(1)	O(1w)-Na(1)-O(7w)a	157.1(1)
O(2w)-Na(1)-O(3w)a	88.4(1)	O(2w)-Na(1)-O(7w)a	101.9(1)
O(3w)a-Na(1)-O(7w)a	84.2(1)	O(11)-Na(2)-O(72)	90.8(1)
O(11)-Na(2)-O(91)	100.0(1)	O(11)-Na(2)-O(1w)	93.0(1)
O(11)-Na(2)-O(2w)	166.2(1)	O(11)-Na(2)-O(4w)	86.8(1)
O(72)-Na(2)-O(91)	104.9(1)	O(72)-Na(2)-O(2w)	99.1(1)
O(72)-Na(2)-O(1w)	175.8(1)	O(72)-Na(2)-O(4w)	93.8(1)
O(91)-Na(2)-O(1w)	72.6(1)	O(91)-Na(2)-O(2w)	86.8(1)
O(91)-Na(2)-O(4w)	159.9(1)	O(1w)-Na(2)-O(2w)	77.5(1)
O(1w)-Na(2)-O(4w)	88.3(1)	O(2w)-Na(2)-O(4w)	83.0(1)
O(11)-Na(3)-O(72)	87.1(1)	O(11)-Na(3)-O(71)	108.1(1)
O(11)-Na(3)-O(5w)	99.8(1)	O(11)-Na(3)-O(6w)	112.0(1)
O(11)-Na(3)-O(8w)	168.9(1)	O(71)-Na(3)-O(72)	84.2(1)
O(71)-Na(3)-O(5w)	97.7(1)	O(71)-Na(3)-O(6w)	139.0(1)
O(71)-Na(3)-O(8w)	70.0(1)	O(72)-Na(3)-O(5w)	171.8(1)
O(72)-Na(3)-O(6w)	89.5(1)	O(72)-Na(3)-O(8w)	103.5(1)
O(5w)-Na(3)-O(6w)	83.8(1)	O(5w)-Na(3)-O(8w)	70.0(1)
O(6w)-Na(3)-O(8w)	72.3(1)	O(53)-Na(4)-O(3w)	91.4(1)
O(53)-Na(4)-O(5w)	175.5(1)	O(53)-Na(4)-O(6w)	101.1(1)
O(53)-Na(4)-O(7w)	92.0(1)	O(53)-Na(4)-O(8w)	103.3(1)
O(3w)-Na(4)-O(5w)	89.8(1)	O(3w)-Na(4)-O(6w)	94.9(1)
O(3w)-Na(4)-O(7w)	82.7(1)	O(3w)-Na(4)-O(8w)	165.2(1)
O(5w)-Na(4)-O(6w)	83.1(1)	O(5w)-Na(4)-O(7w)	83.9(1)
O(5w)-Na(4)-O(8w)	75.6(1)	O(6w)-Na(4)-O(7w)	166.8(1)
O(6w)-Na(4)-O(8w)	81.1(1)	O(7w)-Na(4)-O(8w)	98.0(1)

Symmetry transformations: (a) *x*, *y* + 1, *z*.

**Figure 3:**
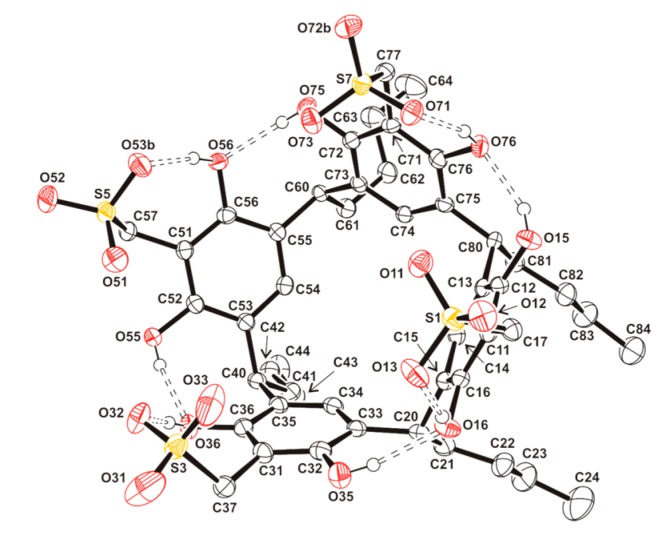
Resorcinarene moiety of **2a** (thermal ellipsoids at the 50% probability level). Methyl, methylene, methine and aromatic hydrogen atoms are omitted for clarity. Relevant hydrogen interactions are showed. Symmetry code: (b) −*x* + 1, −*y*, −*z* + 1.

The resorcinarene moiety shows the four *C*-butyl chains in the lower rim, adopting an *rccc* configuration, which has also been observed for other resorcinarenes [21,22,23,24]. The bond lengths and angles are similar to those found in the reported examples, with C···C diagonal distances of C(14)···C(54) 5.198(6), C(34)···C(74) 5.162(4), C(20)···C(60) 7.152(5) and C(40)···C(80) 7.249(5) Å [20,21,22] (see Figure 3).

On the other hand, the methylsulfonate groups are located in the upper rim of the macrocycle and are involved in O-H···O hydrogen bonding interactions, with a hydroxyl group linked to the same resorcinol ring (Figure 3, Table 3).

**Figure 4:**
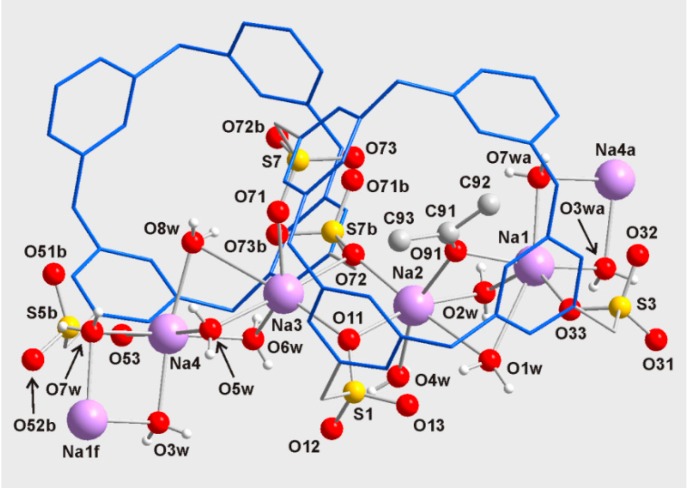
Perspective view of **2a**. Resorcinarenyl units are showed in blue. *C*-butyl chains and non-water hydrogen atoms are omitted for clarity. Symmetry code: (a) *x*, *y* + 1, *z*; (b) −*x* + 1, −*y*, −*z* + 1; (f) *x*, *y* – 1, *z*.

Moreover, the sulfonate moieties are coordinated to sodium atoms, constructing infinite chains of sodium centers, which are bridged by water, acetone or sulfonate fragments, as shown in Figure 4. This system with sodium atoms is reminiscent of those reported for sulfonated calixarenes [25,26].

Focusing on one of these chains, the sodium atom Na(1) is six-coordinate and bonded to one oxygen atom (O(33)) of a sulfonate group (corresponding to S(3)), four water molecules (O(1w), O(2w), O(3w) and O(7w)), and the oxygen atom (O(91)) of an acetone molecule. The bond lengths Na(1)-O are in the range 2.281(3)–2.375(3) Å and compare well with sodium-oxygen coordination bonds [27], except for the long-range interaction Na(1)-O(1w) at 2.863(4) Å. If this long Na(1)-O bond is not considered, the coordination geometry for Na(1) may be described as being distorted trigonal bipyramidal. In this geometry, the atoms O(33), O(2w) and O(7w) occupy the equatorial positions (the sum of angles subtended at the Na(1) center in the equatorial plane results in 360.0°), and the atoms O(91) and O(3w) are in the axial coordination sites, with the O(91)-Na(1)-O(3w) angle at 169.4(1)°. The oxygen atom O(1w) caps the triangular face formed by O(33), O(91) and O(2w).

The sodium atom Na(3) is also six-coordinate through bonding to three oxygen atoms, (O(11), O(71) and O(72)), of the sulfonate groups and three water molecules, (O(5w), O(6w) and O(8w)). The distances Na(3)-O, 2.349(3)–2.514(3) Å, are typical for sodium-oxygen bonds [27], except for the long bond length Na(3)-O(8w) at 2.887(3) Å. The geometry for Na(3) is similar to the coordination described for Na(1). Thus, in a distorted trigonal bipyramidal geometry, the atoms O(72) and O(5w) are located in axial positions, while O(11), O(71) and O(6w) occupy the equatorial plane with a sum of angles resulting in 359.1°. The oxygen atom O(8w) caps the face formed by O(71), O(5w) and O(6w).

**Table 3:** Relevant hydrogen bonds ^a^ for Compound **2a**.

D-H···A	D···A/Å	H···A/Å	D-H···A/°
O(15)-H(15)···O(76)	2.780(4)	1.98(5)	155(4)
O(16)-H(16)···O(13)	2.667(3)	1.86(5)	163(5)
O(35)-H(35)···O(16)	2.823(4)	1.99(5)	170(5)
O(36)-H(36)···O(32)	2.699(4)	1.83(4)	168(4)
O(55)-H(55)···O(36)	2.791(4)	2.02(5)	166(5)
O(56)-H(56)···O(53)b	2.765(3)	1.87(5)	169(4)
O(75)-H(75)···O(56)	2.825(4)	2.04(5)	175(5)
O(76)-H(76)···O(71)	2.721(3)	1.89(5)	164(4)
O(1w)-H(1B)···O(13)	2.665(5)	1.82(4)	162(3)
O(2w)-H(2B)···O(52)c	2.718(4)	1.92(3)	173(3)
O(3w)-H(3A)···O(4w)d	2.756(4)	2.03(3)	149(3)
O(3w)-H(3B)···O(1w)d	2.720(5)	1.98(4)	152(3)
O(4w)-H(4A)···O(12)d	2.756(4)	2.03(3)	149(3)
O(4w)-H(4B)···O(31)e	2.724(5)	1.92(3)	170(2)
O(5w)-H(5A)···O(51)f	2.803(4)	2.03(3)	160(3)
O(5w)-H(5B)···O(32)f	2.849(4)	2.05(4)	166(3)
O(6w)-H(6A)···O(73)b	2.889(4)	2.27(5)	134(4)
O(6w)-H(6B)···O(12)d	2.832(4)	2.03(3)	172(3)
O(7w)-H(7A)···O(51)f	2.728(4)	1.93(5)	166(5)
O(7w)-H(7B)···O(8w)g	2.812(3)	2.01(2)	172(2)
O(8w)-H(8A)···O(73)b	2.796(4)	2.03(4)	158(4)
O(8w)-H(8B)···O(52)f	2.968(4)	2.25(4)	148(3)

^a^ Symmetry transformation: (b) −*x* + 1, −*y*, −*z* + 1; (c) −*x* + 1, −*y* + 1, −*z* + 1; (d) −*x*, −*y*, −*z* + 1; (e) −*x*, −*y* + 1, −*z* + 1; (f) *x*, *y* − 1, *z*; (g) −*x* + 1, −*y* − 1, −*z* + 1. A = acceptor; D = donor. Symmetry operator applied to acceptor atoms.

The sodium atoms Na(2) and Na(4) are also six-coordinate, although the coordination geometry for them is best described as distorted octahedral. Na(2) is linked to two oxygen atoms (O(11) and O(72)) of sulfonate moieties, three water molecules (O(1w), O(2w) and O(4w)) and the oxygen atom (O(91)) of an acetone molecule; whereas the coordination sphere for Na(4) comprises one oxygen (O(53)) of a sulfonate group and five water molecules (O(3w), O(5w), O(6w), O(7w) and O(8w)). The angles O-Na(2)-O and O-Na(4)-O in contiguous positions are in the ranges 72.6(1)–104.9(1)° and 75.6(1)–103.3(1)°, respectively. The distances Na(2)-O and Na(4)-O, spanning 2.327(3)–2.729(4) Å and 2.355(3)–2.495(3) Å, are similar to the other sodium-oxygen bond lengths in the structure.

**Figure 5:**
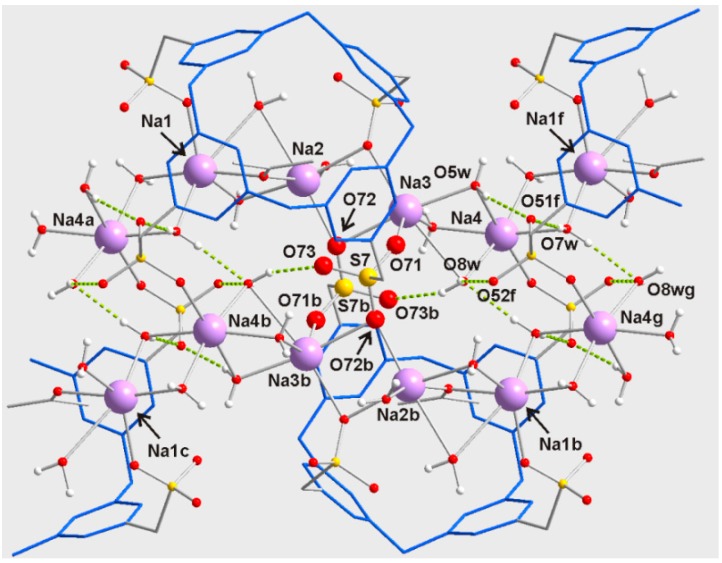
Perspective view of the double chain of sodium atoms for **2a**. Resorcinarenyl cycles are showed in blue. Symmetry transformation: (a) *x*, *y* + 1, *z*; (b) −*x* + 1, −*y*, −*z* + 1; (c) −*x* + 1, −*y* + 1, −*z* + 1; (f) *x*, *y* – 1, *z*; (g) −*x* + 1, −*y* – 1, −*z* + 1.

**Figure 6:**
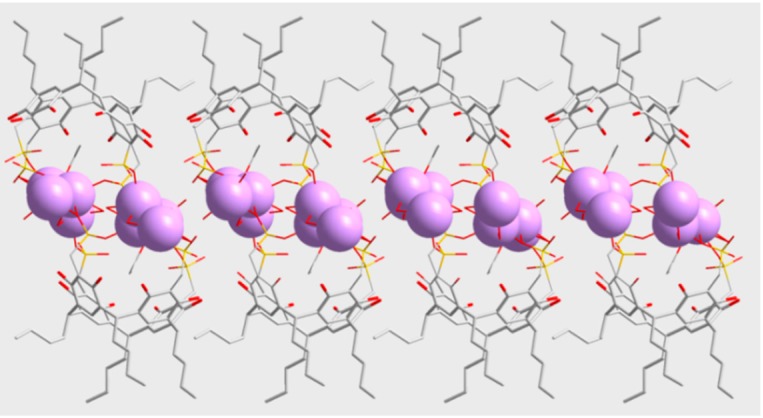
Perspective view of a layer for **2a**. Sodium atoms are showed as purple spheres.

This chain of sodium atoms is connected to another one via two bridging sulfonate groups (corresponding to S(7) and S(7)b) and O-H···O hydrogen bonds (Table 3)), defining a system of two parallel chains (Figure 5). This double chain is covered by the resorcinarene units, providing an infinite one-dimensional array of sodium centers and macrocycles. New O-H···O hydrogen bonding interactions established between the double chains produce layers (Figure 6) that are held in alternating hydrophilic-hydrophobic bilayer packing.

In general, the only difference between the two synthesized compounds is the replacement of the third carbon atom by a sulfur atom in the attached chain of the lower rim of the resorcinarene, but this change appears to be enough to produce differences in the behavior between the sulfonated resorcinarenes. In our work, the differences become evident in our failed attempts of recrystallization of **2b** by similar methods; nevertheless, this is the start of future research focusing on their physicochemical properties and behavior in solution.

## 3. Experimental Section

### 3.1. General Information

FT-IR spectra were recorded in potassium bromide pellets using a Thermo Nicolet IS10 spectrophotometer (Bogotá, Colombia); peaks are reported in cm^–1^. ^1^H- and ^13^C-NMR spectra were recorded on a Bruker Avance (Bogotá, Colombia) (400 MHz for ^1^H-NMR and 100 MHz for ^13^C-NMR) spectrometer in CD_3_OD and/or D_2_O; the chemical shifts are given in δ units (ppm). Mass spectra were performed on an Agilent Infinite 1260 mass spectrometer (Bogotá, Colombia), QToF 6520 detector. Analytical thin layer chromatography (TLC) was determined on aluminum sheets precoated with silica gel (Merck, Kieselgel 60 PF_254_). Visualization was accomplished by UV light. The elemental analysis for Carbon, Hydrogen and Sulphur was carried out using a Thermo Flash 2000 Elemental Analyzer (Bogotá, Colombia). The thermogravimetric analysis (TG) was done using a Netzsch STA 409 thermobalance (Bogotá, Colombia) with a sample weight between 14 and 28 mg, over a temperature range of 20–900 °C and with a heating rate of 10 °C/min. The measurements were carried out in nitrogen atmosphere (flow rate 16.66 mL/min) by using an alumina crucible.

### 3.2. General Synthetic Method for Resorcinarenes **1a** and **1b**

This was performed according to the procedure given by Hogberg [17]. To a solution of resorcinol (0.10 mol) and the respective aldehyde (valeraldehyde or 3-methylthiopropanaldehyde, 0.10 mol), in a 1:1 ethanol/water mixture (40 mL), concentrated hydrochloric acid (10 mL) was added. The reaction mixture was stirred at 75 °C for 1 h, afterwards cooled in an ice bath and, finally, filtered. The precipitate was recrystallized in water, washed and dried at 60 °C for 48 h.

*C-Tetra(butyl)resorcinarene* (**1a**): Pale yellow powder. Yield 87%; IR (KBr, υ cm^–1^): 3314 (O-H), 1194 (C-O); ^1^H-NMR (δ, DMSO-*d*_6_): 0.90 (t, 12H, *J* = 8.0 Hz, -CH_3_), 1.23 (m, 16H, CH_2_CH_2_), 2.11 (q, 8H, *J* = 8.0 Hz, CH_2_), 4.23 (t, 4H, *J* = 8.0 Hz, CH), 6.15 (s, 4H, ArH, *ortho* to OH), 7.26 (s, 4H, ArH, *meta* to OH), 8.94 (s, 8H, OH). ^13^C-NMR (δ, DMSO-*d*_6_): 16.0 (CH_3_), 25.8 (CH_2_), 33.5 (CH_2_), 36.5 (CH_2_), 49.1 (CH), 111.2, 126.9, 129.2, 151.0 (C-Ar); ESI-MS *m/z* 712.90 ((M−H)^−^ calcd. for C_44_H_56_O_8_ 712.91). Anal. calcd. for (molecular formula, C_44_H_56_O_8_): C = 74.13, H = 7.92; found: C, 74.02, and H, 7.98.

*C-Tetra(2-(methylthio)ethyl)resorcinarene* (**1b**): Pale yellow powder. Yield 82%; IR (KBr, υ·cm^−1^): 3330 (O-H), 1160 (C-O), 605 (C-S); ^1^H-NMR (δ, CD_3_OD): 2.13 (s, 12H, CH_3_S), 2.47 (m, 16H, CH_2_CH_2_), 4.50 (m, 4H, CH), 6.39 (s, 4H, ArH, *ortho* to OH), 7.24 (s, 4H, ArH, *meta* to OH). ^13^C-NMR (δ, CD_3_OD): 15.8 (CH_3_), 33.7 (CH_2_), 34.8 (CH_2_), 48.1 (CH), 110.5, 127.3, 129.7, 151.0 (C-Ar); ESI-MS *m/z* 784.06 ((M−H)^−^ calcd. for C_40_H_48_O_8_S_4_ 784.06). Anal. calcd. for (molecular formula, C_40_H_48_O_8_S_4_): C = 61.20, H = 6.16 and S = 16.34; found: C, 61.06, H, 6.22, and S, 16.32.

### 3.3. General Method for Sulfonation of Resorcinarenes

This was performed according to the procedure specified by Kazakova *et al*. [19]. The product (**1a** or **1b**, 0.01 mol) was dissolved in a mixture containing formaldehyde (37%, 4.1 g, 0.05 mol), sodium sulfide (6.3 g, 0.05 mol) and water (30 mL). After a stirring period of 4 h at 90–95 °C, the reaction mixture was cooled to room temperature and neutralized with hydrochloric acid. Then, acetonitrile was added in order to precipitate the sulfonated resorcinarene. The resulting product was recrystallized in acetonitrile, washed with the same solvent and dried at 80 °C under vacuum for 24 h.

*Tetrasodium 5,11,17,23-tetrakissulfonatemethylen-2,8,14,20-tetra(butyl)resorcinarene* (**2a**): Pale yellow powder. Yield 81%; IR (KBr, υ cm^−1^): 3421 (O-H), 2929 (C-H), 1609 (C=C, Ar), 1215 (C-O), 1044 (S=O), 773 (S-O); ^1^H-NMR (δ, CD_3_OD): 1.09 (t, 12H, *J* = 7.6 Hz, CH_3_), 1.45 (m, 8H, CH_2_), 1.60 (m, 8H, CH_2_), 2.35 (q, 8H, *J* = 7.6 Hz, CH_2_), 4.41 (s, 8H, CH_2_), 4.60 (t, 4H, *J* = 7.6 Hz, CH), 7.39 (s, 4H, ArH). ^13^C-NMR (δ, CD_3_OD): 16.1 (CH_3_), 25.6 (CH_2_), 32.7 (CH_2_), 37.0 (CH_2_), 50.1 (CH), 54.8 (CH_2_), 112.1, 127.2, 129.0, 153.1 (C-Ar); ESI-MS *m/z* 1153.23 ((M−Na)^−^ calcd. for C_48_H_60_Na_3_O_20_S_4_ 1153.20). Anal. calcd. for (molecular formula, C_48_H_60_Na_4_O_20_S_4_ + 3H_2_O): C = 46.82, H = 5.40 and S = 10.42; found: C, 46.65, H, 5.45, and S, 10.39.

*Tetrasodium 5,11,17,23-tetrakissulfonatemethylen-2,8,14,20-tetra(2-(methylthio)ethyl)resorcinarene* (**2b**): Pale yellow powder. Yield 65%. IR (KBr, υ cm^−1^): 3442 (O-H), 2918 (C-H), 1612 (C=C, Ar), 1190 (C-O), 1045 (S=O), 757 (S-O), 605 (C-S). ^1^H-NMR (δ, D_2_O): 2.09 (s, 12H, CH_3_S), 2.43–2.49 (m, 16H, CH_2_CH_2_), 4.20 (s, 8H, CH_2_), 4.39 (m, 4H, CH), 7.19 (s, 4H). ^13^C-NMR (δ, D_2_O): 15.8 (CH_3_), 32.8 (CH_2_), 35.8 (CH_2_), 47.8 (CH), 52.6 (CH_2_), 110.5, 127.3, 129.7, 151.0 (C-Ar); ESI-MS *m/z* 1226.35 ((M−Na)^–^ calcd. for C_44_H_52_Na_3_O_8_S_4_ 1226.35). Anal. calcd. for (molecular formula, C_44_H_52_Na_4_O_20_S_8_ + 3H_2_O): C = 40.55, H = 4.49 and S = 19.68; found: C, 40.61, H, 4.53, and S, 19.64.

### 3.4. X-ray Crystallography

Crystals of **2a** were obtained from a water/acetone (1:1) mixture by slow evaporation at room temperature. The crystals were removed from the vial and covered with a layer of a viscous perfluoropolyether (Fomblin Y). A suitable crystal was selected with the help of a microscope, mounted on a cryoloop and placed in the low-temperature nitrogen stream of the diffractometer. The intensity datasets were collected at 200 K on a Bruker-Nonius KappaCCD diffractometer equipped with an Oxford Cryostream 700 unit. Crystallographic data are presented in Table 4.

The structure was solved, using the WINGX package [28], y direct methods (SHELXS-2013) [29] and refined by least-squares against F^2^ (SHELXL-2014) [29]. Compound **2a** crystallized with eight molecules of water and two molecules of acetone. All non-hydrogen atoms were anisotropically refined. Methyl, methylene, methine and aromatic hydrogen atoms were positioned geometrically and refined by using a riding model in the last cycles of refinement, whereas hydroxyl and water molecules’ hydrogen atoms were isotropically refined. SADI restraints were employed for the water molecules, except the molecule corresponding to O(1w), which was treated with DFIX instruction. Moreover, U*iso* or hydrogen atoms H(1A)) and H(1B), linked to O(1w), were fixed with a value of 0.05. CCDC 1050083 contains the supplementary crystallographic data for the present paper [30].

**Table 4:** Crystallographic data for compound **2a**.

Crystal Parameters	Data/Values
CCDC ^a^ deposition number	1050083
Empirical formula	C_54_H_88_Na_4_O_30_S_4_
Moiety formula	C_51_H_82_Na_4_O_29_S_4_, C_3_H_6_O
Formula weight	1437.44
Temperature	200(2) K
Wavelength	0.71073 Å
Crystal system	Triclinic
Space group	P-1
Unit cell dimensions	*a* = 12.055(2) Å, α = 80.28(1)°
	*b* = 12.384(1) Å, β = 88.26(1)°
	*c* = 23.563(3) Å, γ = 72.81(1)°
Volume	3311.7(8) Å^3^
Z	2
D_cal_	1.442 g·cm^−3^
Absorption coefficient	0.256 mm^−1^
F(000)	1520
Crystal size	0.16 × 0.10 × 0.10 mm^3^
range	3.06° to 27.50°
Index ranges (h, k, l)	−15 to 15
	−16 to 16
	−30 to 30
Reflections collected	99438
Unique data	15,183, R(int) = 0.107
Observed data (I > 2σ(I))	8986
Goodness-of-fit on F^2^	1.040
Final R indices (I > 2σ(I))	R1 = 0.066, wR2 = 0.136
R indices (all data)	R1 = 0.134, wR2 = 0.167
Largest diffraction peak and hole	1.219 and −0.572 e·Å^−3^

^a^ Cambridge Crystallographic Data Centre.

## 4. Conclusions

In the present investigation, two novel sulfonated resorcinarenes were prepared in good yields and high purity through reactions of cyclocondensation and sulfonation using resorcinol and aldehydes (valeraldehyde or 3-methylthiopropanaldehyde) as raw materials. NMR data suggest that the preferred conformation in solution of **2a** and **2b** is *rccc* (cone conformation), and thermogravimetric analysis revealed that the number of water molecules in both compounds is three in the amorphous solid state. The X-ray structure of **2a** was in good agreement with the NMR data and corroborates the fact that only one *rccc* conformation is present in the crystalline solid. Finally, in the crystal packing of **2a**, the molecules come together to form a linear array based on sodium centers and sulfonated resorcinarenes with a capsule motif, and the bilayer is stabilized in the extended structure.
